# Isolation and Evaluation of the Enantiospecific Antitubercular Activity of a Novel Triazole Compound

**DOI:** 10.3797/scipharm.1308-15

**Published:** 2013-10-21

**Authors:** Radha Shekar, Barij Nayan Sinha, Arindam Mukhopadhya, Mariam S. Degani

**Affiliations:** 1 Lotus Labs Pvt. Ltd, Bangalore, India.; 2 Birla Institute of Technology, Ranchi, India.; 3 Lotus Clinical Research Academy, Bangalore, India.; 4 Institute of Chemical Technology, Mumbai, India.

**Keywords:** Chiral Separation, Preparative Chiral Chromatography, Triazoles, Antitubercular Compounds, REMA testing

## Abstract

Cyclohex-3-enyl(5-phenyl-4*H*-1,2,4-triazol-3-yl)methanol (MSDRT 12) is a novel triazole-based antitubercular compound with two chiral centers. To evaluate the enantiospecific antitubercular activity, the four stereoisomers were isolated using preparative chiral chromatography and the individual stereoisomers were evaluated using the resazurin microtiter assay method (REMA) and a microbroth dilution technique against the *Mycobacterium tuberculosis* H37Rv strain. Isomer III of MSDRT 12 was found to be the most potent with a minimum inhibitory concentration (MIC) of 0.78 μg/mL, Isomer II had a MIC of 12.5 μg/mL, and isomers I and IV showed no activity. The diastereomeric mixture of MSDRT 12 showed a MIC of 3.125 μg/mL and isoniazid, used as the standard drug, showed a MIC of 0.4 μg/mL. This confirms the necessity of screening individual enantiomers for their pharmacological activity early in the discovery phase to identify the most potent isomer for further development efforts.

## Introduction

Molecular chirality is a fundamental consideration in drug discovery to understand and describe biological targets as well as to design effective pharmaceutical agents [1]. The regulatory guidance for chiral drugs published by the United Stated Food and Drug Administration (USFDA) in 1992 requires that the manufacture and control of a chiral drug should ensure its stereoisomeric composition with respect to identity, strength, quality, and purity [2]. Racemates are considered mixtures of compounds with different pharmacological activities, and the estimation of pharmacokinetic parameters based on ‘total’ drug concentrations following administration of a racemate is therefore of limited value [3]. It is necessary to develop quantitative assays for individual enantiomers and to determine the main pharmacologic activities of each enantiomer.

Enantioselective chromatography is now playing an increasing role not only as an analytical tool for chiral analysis, but also as a preparative technique to obtain pure enantiomers from racemates quickly from a wide diversity of chemical structures [4, 5]. Asymmetric synthesis in early phases of drug discovery is not preferred as it is expensive, time-consuming, and can generate only one of the enantiomers. Preparative chiral chromatography on chiral stationary phases (CSPs), however, is widely used as it is efficient, easily scalable, and can generate all the stereoisomers with high purity [6–10].

Tuberculosis is a major health problem worldwide, with approximately two million people dying annually from the disease. The long-standing current drug regimen, the emergence of drug-resistant strains, and HIV co-infection have resulted in a resurgence in research efforts to address the urgent need for new anti-tuberculosis drugs [11]. A number of new potential anti-tuberculosis drug candidates with novel modes of action are being developed and the shikimate pathway represents an attractive target for the development of new antitubercular agents since it is present and essential in bacteria, but absent in mammals [12]. Hybrid compounds containing an alicyclic scaffold which is a known dehydroquinase inhibitor, and a substituted heterocyclic moiety like triazole which is a known shikimate kinase inhibitor, were synthesised as novel antitubercular agents [13].

**Fig. 1:**
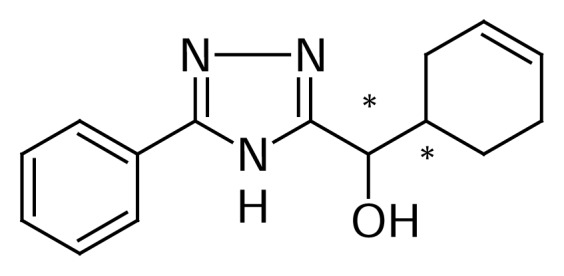
Structure of Cyclohex-3-enyl(5-phenyl-4*H*-1,2,4-triazol-3-yl)methanol (MSDRT 12)

**Fig. 2:**
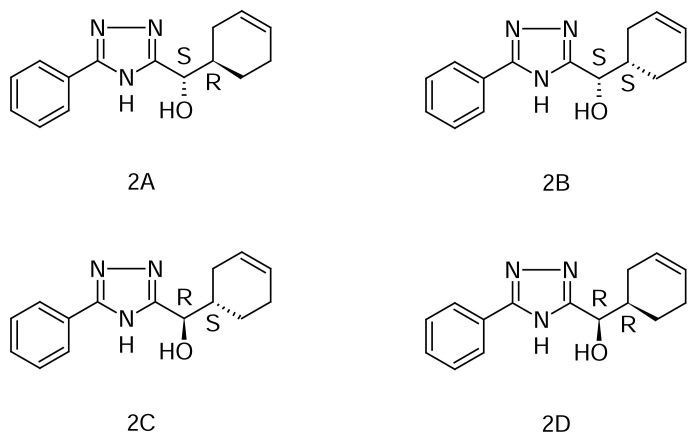
Stereoisomers of MSDRT 12 2A: (*S*)-(1*R*)-Cyclohex-3-en-1-yl(5-phenyl-4*H*-1,2,4-triazol-3-yl)methanol 2B: (*S*)-(1*S*)-Cyclohex-3-en-1-yl(5-phenyl-4*H*-1,2,4-triazol-3-yl)methanol 2C: (*R*)-(1*S*)-Cyclohex-3-en-1-yl(5-phenyl-4*H*-1,2,4-triazol-3-yl)methanol 2D: (*R*)-(1*R*)-Cyclohex-3-en-1-yl(5-phenyl-4*H*-1,2,4-triazol-3-yl)methanol

Cyclohex-3-enyl(5-phenyl-4*H*-1,2,4-triazol-3-yl)methanol (MSDRT 12) (Figure 1), a novel synthetic triazole compound, has been characterized using ^1^H-NMR and IR spectroscopy and mass spectroscopy [13]. The compound MSDRT 12 showed good *in vitro* anti-tubercular activity in the resazurin microtiter assay [14–17]. MSDRT 12 has two chiral centers with four isomers as shown in Figure 2.

This paper describes the separation of the four stereoisomers by preparative chiral chromatography and the evaluation of the enantiospecific antitubercular activity of each stereoisomer.

## Experimental

### Materials

The compound MSDRT 12 has been synthesized at the Institute of Chemical Technology (ICT), Mumbai, India. The HPLC grade solvents, *n*-hexane, isopropyl alcohol, diethylamine, dimethylsulfoxide, and methanol, were procured from Merck, whereas ethanol was from Tedia. Middlebrook medium, albumin-dextrose catalase (ADC), and 96-well non-treated plates used for the REMA test were supplied by HiMedia. Resazurin was supplied by SRL, India. The *Mycobacterium tuberculosis* H37Rv strain (Mtb) was maintained at the Radiation Medicine Centre, Mumbai.

### Equipment

The K-Prep system with a flow range of 0–100 mL/minute manufactured by YMC was used for preparative chromatography. A Sanyo carbon dioxide incubator was used for incubating the plates for REMA testing.

### Isolation of Four Stereoisomers of MSDRT 12

Compound MSDRT 12 (diastereomeric mixture) was supplied by ICT, Mumbai, India. Using the chiral analytical column Chiralpak ID (4.6 mm i.d. x/250 mm, particle size 5μ) and a mobile phase of *n*-hexane:isopropyl alcohol: ethanol: diethylamine (60:35:05:0.1 v/v/v/v) at a flow rate of 1 mL/min with detection at 230 nm, it was established that four stereoisomers (identified as Isomer I, Isomer II, Isomer III, and Isomer IV based on the order of elution with retention times of 8.6, 9.6, 13.0, and 19.2 minutes, respectively) were in the ratio 37:13:37:13 in MSDRT 12. This analytical method could not be directly scaled up for preparative chromatography because of poor resolution between isomers I and II due to the increased loading in the preparative scale. Hence, a two-step procedure was developed for preparative scale chromatography.

**Fig. 3:**
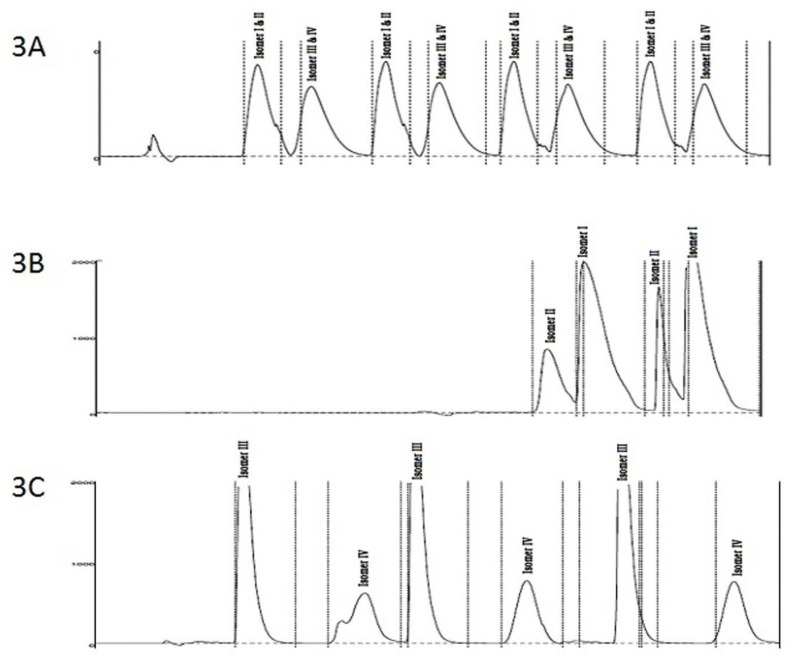
Preparative loading chromatogram showing the three-step purification for separation of the four stereoisomers 3A: MSDRT 12 (diastereomeric mixture) 3B: Isomers I and II 3C: Isomers III and IV

In the first step, fraction 1 (consisting of isomers I and II) was separated from the second fraction (consisting of isomers III and IV) by injecting 10 mL of a solution of MSDRT 12 in methanol (2.5 mg/mL) on a Chiralpak IE (250 × 30mm, 5 μ) column at 25°C using the mobile phase *n*-hexane:isopropyl alcohol:diethylamine (70: 30: 0.1) at a flow rate of 42 mL/minute and detected at 250 nm (Figure 3A). Each fraction was evaporated to dryness using a roto-evaporator at a temperature of 30°C and reconstituted in methanol (2.5 mg/mL) for further separation.

In the second step, separation of isomers I and II was achieved by injecting 6 mL of fraction 1 into a preparative column, Chiralpak IA (250 × 20mm, 5μ particle size) using *n*-hexane:isopropyl alcohol:diethylamine (70:30:0.1 v/v/v) as the mobile phase with a flow rate of 18 mL/minute. The column temperature was maintained at 25°C and each isomer was detected at 250 nm (Figure 3B).

Similarly, isomers III and IV were further separated by injecting 10 mL of fraction 2 into a preparative column, Chiralpak AY-H (250 × 30 mm, 5 μ) at 25°C using the mobile phase *n*-hexane: isopropyl alcohol:methanol:diethylamine (50:40:10:0.1 v/v/v/v) at a flow rate of 42 mL/min. The column temperature was maintained at 25°C and each isomer was detected at 250 nm (Figure 3C).

### Resazurinmicrotitre Assay (REMA)

All five compounds (Isomers I, II, III, IV, and MSDRT 12) were tested against *Mycobacterium tuberculosis* H37Rv strain to determine the minimum inhibitory concentration (MIC) in the broth microdilution assay. The MIC is defined as the minimum concentration of a compound required for the complete inhibition of bacterial growth, as indicated by the colour change of resazurin (from blue to pink in case of growth). A serial dilution (from 50 μg/mL to 0.1 μg/mL) of each of the five compounds (Isomer I, Isomer II, Isomer III, Isomer IV, and MSDRT 12 (diastereomeric mixture)) was prepared in a volume of 200 μL containing a homogenous *Mycobacterium tuberculosis* H37Rv culture suspension 5×10^4^ Mtb in Middlebrook media. In case of the control, an equivalent amount of dimethyl sulfoxide was added to the plate. The plates were incubated at 37°C for 7 days in a carbon dioxide incubator. Resazurin dye (0.02% in Middlebrook medium) was added after 7 days and the plates were incubated for 48 hours. The MIC was determined by visual inspection of the dye colour (blue to pink). Isoniazid was used as the standard drug.

### Purity of the Stereoisomer

**Fig. 4:**
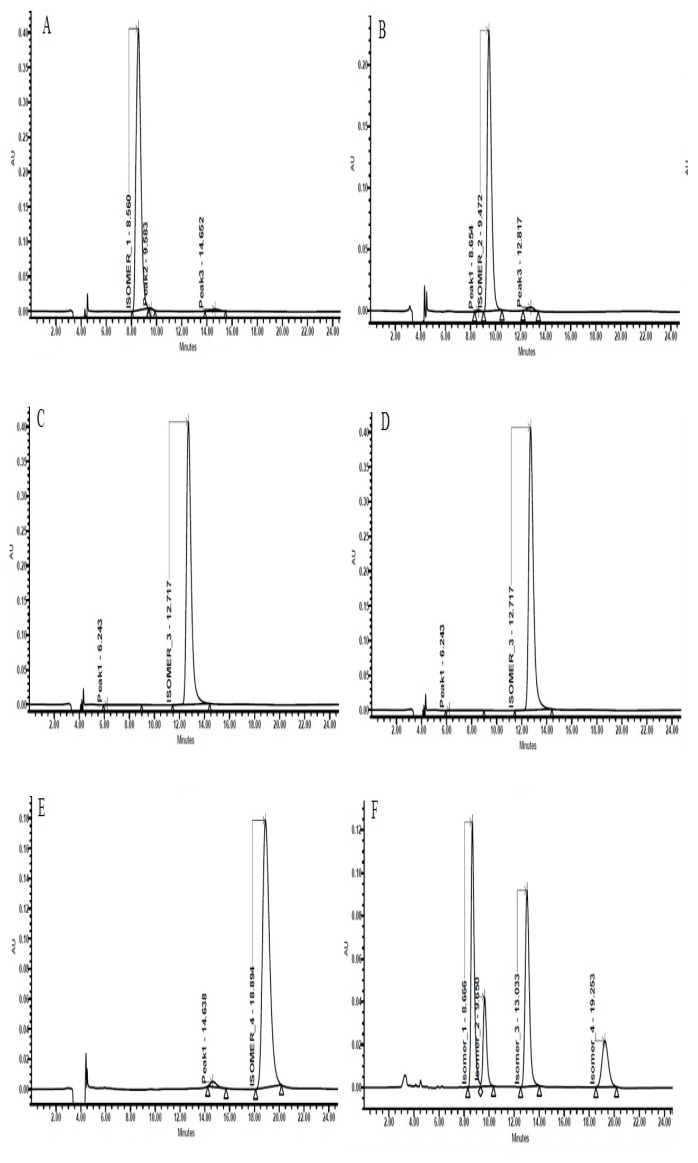
Representative chromatograms of Isomer I (A), Isomer II (B), Isomer III (C), Isomer IV (D), Racemic mixture of MSDRT 12 (E)

**Fig. 5:**
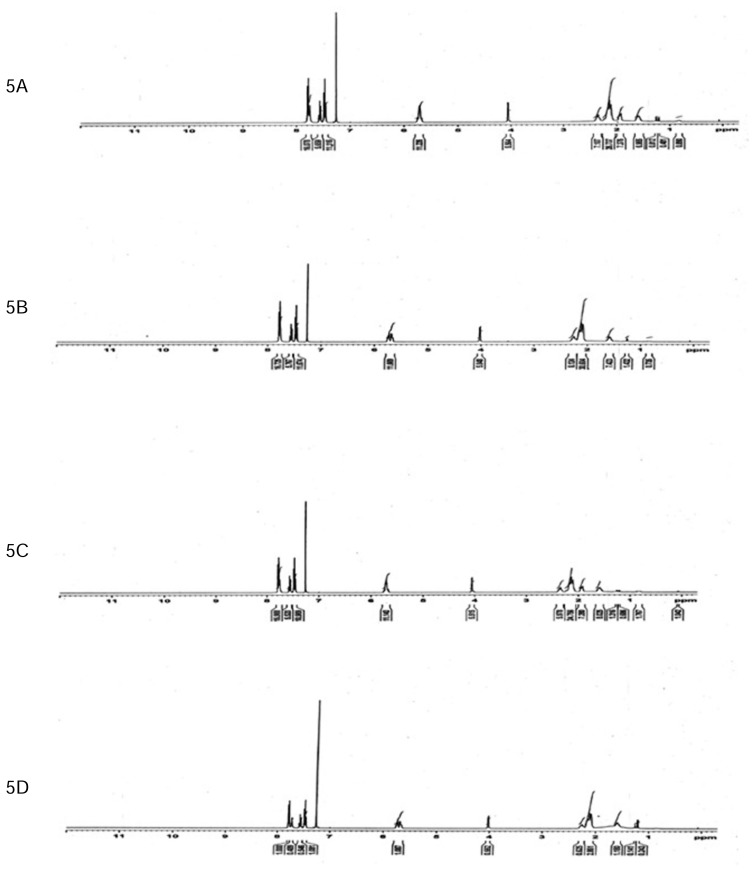
^1^H NMR of Isomer I (5A), Isomer II (5B), Isomer III (5C), and Isomer IV (5D) of MSDRT 12

**Fig. 6:**
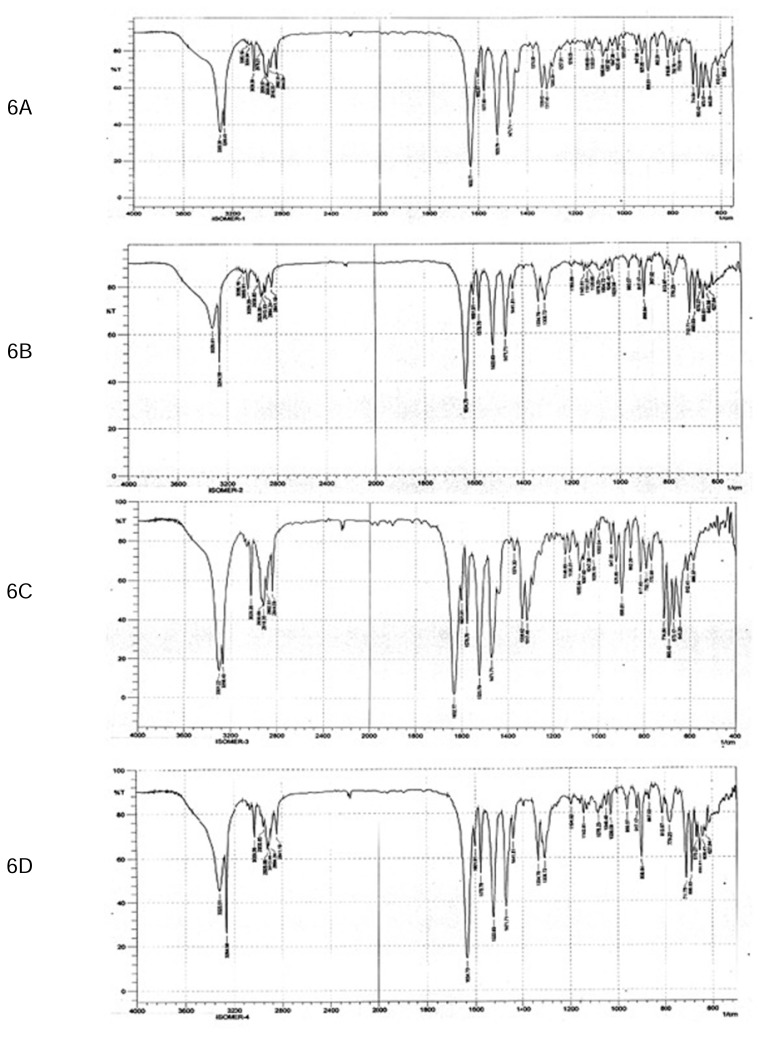
IR spectra of Isomer I (6A), Isomer II (6B), Isomer III (6C), and Isomer IV (6D) of MSDRT 12

**Fig. 7:**
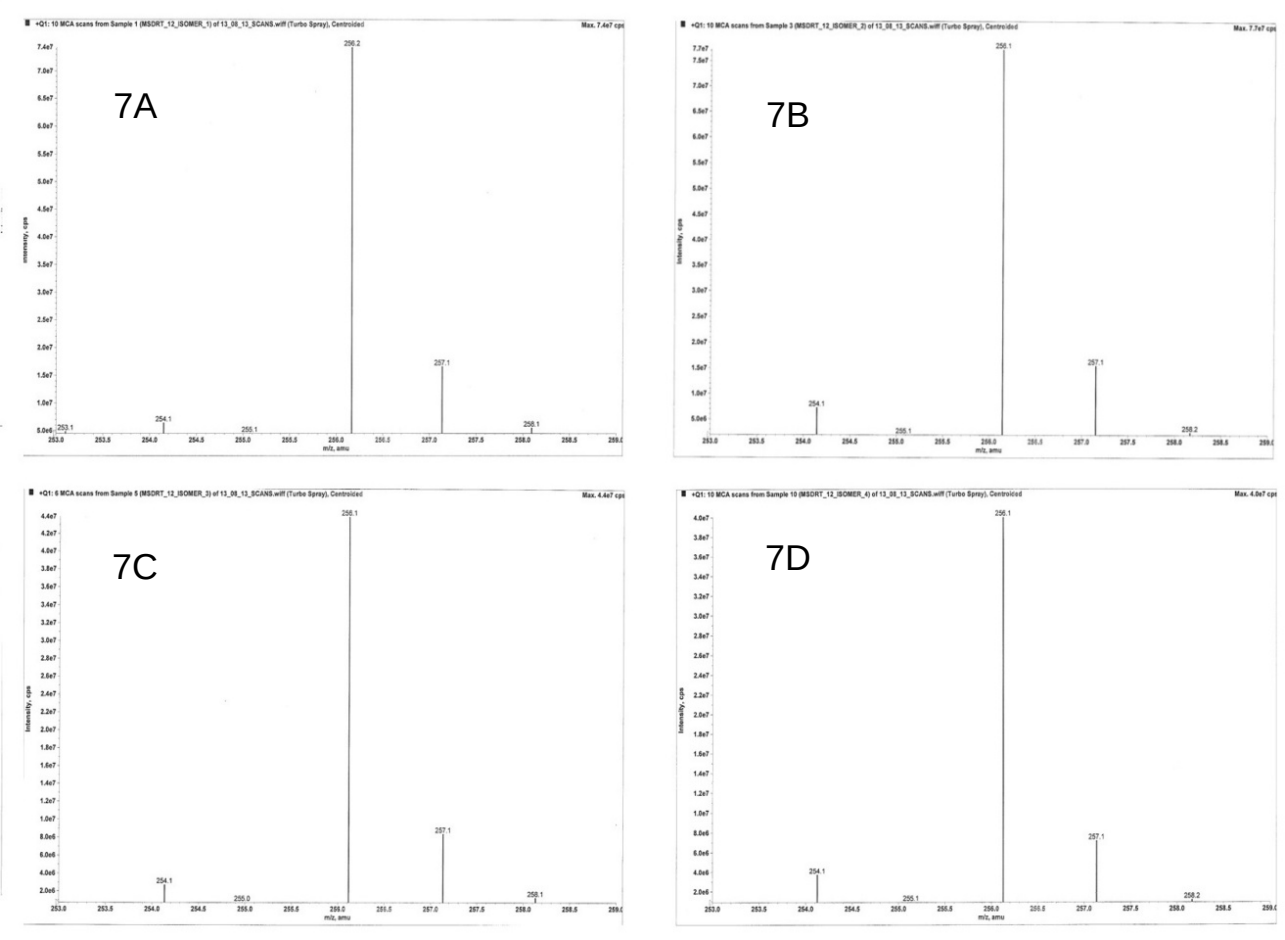
Mass spectra of Isomer I (7A), Isomer II (7B), Isomer III (7C), and Isomer IV (7D) of MSDRT 12

The purity of each stereoisomer was confirmed using a validated chiral analytical method. Enantiopurities were 98.7, 99.8, 99.1, 99.5% for isomers I, II, III, and IV, respectively. To ensure the stability of the purified isomers, each isomer was analysed initially and also after evaporation in the roto evaporator at 30°C followed by reconstitution in methanol. Lack of interconversion and no significant increase (less than 0.3%) in impurity levels confirmed the stability of each pure stereoisomer (Figure 4). The four stereoisomers of MSDRT 12 were further characterised by ^1^H NMR (Figure 5), IR Spectroscopy (Figure 6), and mass spectra (Figure 7).

## Results and Discussion

Thus by using preparative chiral chromatography, it was possible to rapidly isolate all four stereoisomers with the high degree of enantiopurity required for the evaluation of the antitubercular activity.

The MIC of the pure isomers and the synthesized MSDRT 12 (diastereomeric mixture) is detailed in Table 1. Isomer III exhibited good activity with an MIC of 0.78 μg/mL. Isomers I and IV did not show any activity, while Isomer II had a MIC of 12.5 μg/mL, and isoniazid used as the standard drug showed a MIC 0.4 μg/mL. Isomer III was found to be four times more potent when compared to MSDRT 12 (diastereomeric mixture containing the four isomers in a ratio 37:13:37:13).

## Conclusion

Chiral separation and enantiospecific pharmacological activity in early discovery stages help in making a decision whether the chiral compound under investigation should be developed as a racemate or as a single enantiomer. Development of a single pharmacologically active isomer will eliminate/reduce the toxicity due to the unwanted isomers. MSDRT 12 is a newly synthesised, novel, alicyclic triazole compound with promising antitubercular activity against the *Mycobacterium tuberculosis* H37Rv strain. Four stereoisomers of MSDRT 12 were separated and tested individually against the *Mycobacterium tuberculosis* H37Rv strain. Isomer III showed it was significantly more potent when compared to other isomers or the racemate MSDRT 12. Isomer III should therefore be considered as a potential lead compound for further development as an antitubercular drug.

## Figures and Tables

**Tab. 1:** Biological evaluation data for the pure stereoisomers I, II, III, IV, and MSDRT 12

Compound	MIC μg/ml	Enantiopurity/Ratios
Isomer I	No activity	98.7
Isomer II	12.5	99.8
Isomer III	0.78	99.1
Isomer IV	No activity	99.5
MSDRT 12 (Diastereomeric mixture)	3.125	37:13:37:13
Isoniazid	0.4	–
